# Diagnostic accuracy of color-coded virtual noncalcium reconstructions derived from portal venous phase dual-energy CT in the assessment of lumbar disk herniation

**DOI:** 10.1007/s00330-021-08354-2

**Published:** 2021-11-24

**Authors:** Vitali Koch, Moritz H. Albrecht, Leon D. Gruenewald, Ibrahim Yel, Katrin Eichler, Tatjana Gruber-Rouh, Renate M. Hammerstingl, Iris Burck, Julian L. Wichmann, Leona S. Alizadeh, Thomas J. Vogl, Lukas Lenga, Christoph Mader, Simon S. Martin, Silvio Mazziotti, Tommaso D’Angelo, Christian Booz

**Affiliations:** 1 Division of Experimental Imaging, Department of Diagnostic and Interventional Radiology, University Hospital Frankfurt, Theodor-Stern-Kai 7, 60590 Frankfurt am Main, Germany; 2 Department of Diagnostic and Interventional Radiology, University Hospital Frankfurt, Theodor-Stern-Kai 7, 60590 Frankfurt am Main, Germany; 3 Department of Biomedical Sciences and Morphological and Functional Imaging, University Hospital Messina, Via Consolare Valeria 1, 98100 Messina, Italy

**Keywords:** Herniated disk, Computed tomography, X-ray, Spine, Contrast agent, Virtual noncalcium reconstructions

## Abstract

**Objectives:**

To investigate the diagnostic accuracy of color-coded contrast-enhanced dual-energy CT virtual noncalcium (VNCa) reconstructions for the assessment of lumbar disk herniation compared to unenhanced VNCa imaging.

**Methods:**

A total of 91 patients were retrospectively evaluated (65 years ± 16; 43 women) who had undergone third-generation dual-source dual-energy CT and 3.0-T MRI within an examination interval up to 3 weeks between November 2019 and December 2020. Eight weeks after assessing unenhanced color-coded VNCa reconstructions for the presence and degree of lumbar disk herniation, corresponding contrast-enhanced portal venous phase color-coded VNCa reconstructions were independently analyzed by the same five radiologists. MRI series were additionally analyzed by one highly experienced musculoskeletal radiologist and served as reference standard.

**Results:**

MRI depicted 210 herniated lumbar disks in 91 patients. VNCa reconstructions derived from contrast-enhanced CT scans showed similar high overall sensitivity (93% vs 95%), specificity (94% vs 95%), and accuracy (94% vs 95%) for the assessment of lumbar disk herniation compared to unenhanced VNCa images (all *p* > .05). Interrater agreement in VNCa imaging was excellent for both, unenhanced and contrast-enhanced CT (*κ* = 0.84 vs *κ* = 0.86; *p* > .05). Moreover, ratings for diagnostic confidence, image quality, and noise differed not significantly between unenhanced and contrast-enhanced VNCa series (all *p* > .05).

**Conclusions:**

Color-coded VNCa reconstructions derived from contrast-enhanced dual-energy CT yield similar diagnostic accuracy for the depiction of lumbar disk herniation compared to unenhanced VNCa imaging and therefore may improve opportunistic retrospective lumbar disk herniation assessment, particularly in case of staging CT examinations.

**Key Points:**

*• Color-coded dual-source dual-energy CT virtual noncalcium (VNCa) reconstructions derived from portal venous phase yield similar high diagnostic accuracy for the assessment of lumbar disk herniation compared to unenhanced VNCa CT series (94% vs 95%) with MRI serving as a standard of reference.*

*• Diagnostic confidence, image quality, and noise levels differ not significantly between unenhanced and contrast-enhanced portal venous phase VNCa dual-energy CT series.*

*• Dual-source dual-energy CT might have the potential to improve opportunistic retrospective lumbar disk herniation assessment in CT examinations performed for other indications through reconstruction of VNCa images.*

## Introduction

Degenerative intervertebral changes of the lumbar spine with concomitant herniation of lumbar disks represent one of the most common reasons for lower back pain and the principal cause for spinal surgery [1, 2]. Considering that the vast majority of people sustain an episode of lower back pain once during their lifetime, early detection and treatment initiation are essential to avoid high treatment costs and poor outcome [3, 4].

According to the current clinical guidelines of the North American Spine Society (NASS) [5], MRI represents the most accurate and preferred diagnostic imaging modality given its ability for accurate demarcation of the intervertebral disk from surrounding cerebrospinal fluid [6]. Despite these advantages, MRI is known to have several absolute and relative contraindications, including patients with claustrophobia, pacemakers, or other metal implants [7]. Additionally, MRI availability is usually restricted to regular working hours. Thus, many patients may benefit from an alternative method for assessing disk herniation [5, 8, 9].

Over the last decades, the dual-energy CT technique has become increasingly popular as a widely spread diagnostic modality in miscellaneous fields of musculoskeletal and oncological imaging, based on material characterization and differentiation by applying different energy spectra [8, 10–20]. A range of recently developed musculoskeletal applications allows for more detailed visualization of spinal alterations compared to conventional CT [8, 10, 21]. In this context, a novel dedicated postprocessing approach using colored virtual noncalcium (VNCa) reconstructions derived from unenhanced images has been shown to yield substantially higher diagnostic performance and confidence for depicting lumbar disk herniation compared to standard grayscale CT [13, 14, 22]. Given the fact that most CT examinations are performed using contrast agent in the context of tumor staging and vascular or infectious diseases, it remains unknown if the results of Booz et al. may also be applicable for VNCa imaging derived from contrast-enhanced portal venous phase CT series [14].

We hypothesized that the recently published VNCa postprocessing algorithm for color-coded visualization of lumbar disk herniation derived from unenhanced CT scans may also enable the accurate depiction of lumbar disk herniation in VNCa reconstructions derived from contrast-enhanced portal venous phase CT. Thus, the purpose of this study was to evaluate whether intravenously injected iodine contrast agent significantly affects the diagnostic accuracy of VNCa imaging for the assessment of lumbar disk herniation.

## Methods

This retrospective single-center study was approved by the institutional review board with a waiver for written informed consent.

### Patient selection

**Fig. 1:**
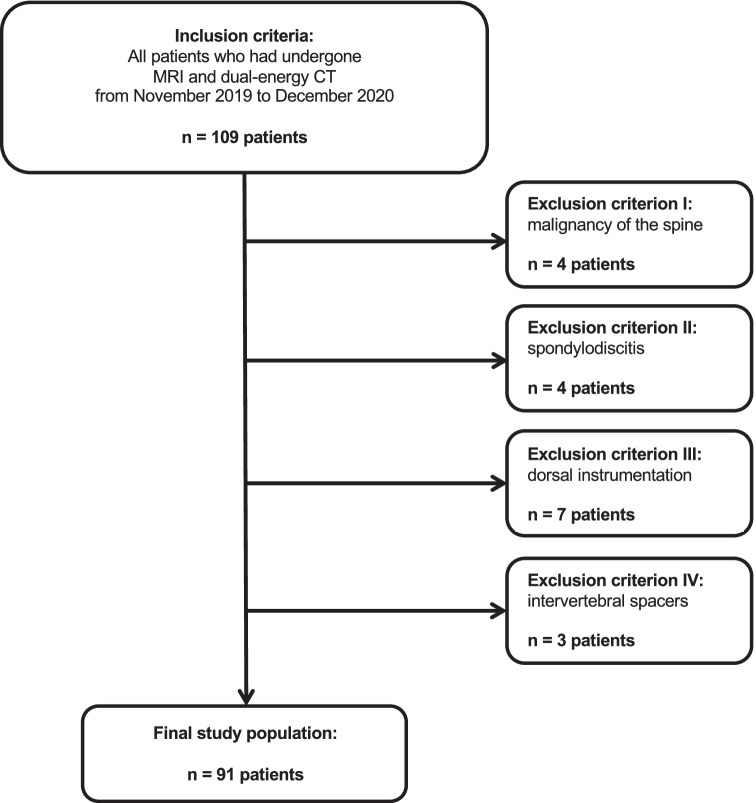
Flowchart of patient inclusion

A total of 109 consecutive patients who had undergone routinely performed third-generation dual-source dual-energy CT and 3.0-T MRI of the lumbar spine between November 2019 and December 2020 were candidates for study inclusion. To identify suitable patients, the picture archiving and communication system (Centricity, version 4.2; GE Healthcare) was searched for the following terms: “lumbar disk herniation”, “herniated disk”, “disk protrusion”, “disk extrusion”, and “disk sequestration”. The search resulted in more than 200 initial hits, of which 109 patients were found to have undergone both CT and MRI at our institution. In this context, a considerable part of the image series consisted of both unenhanced and contrast-enhanced scans. As we have experienced some limitations regarding virtual non-contrast CT series in terms of image quality and artifacts, we regularly perform a true non-contrast scan in each examination if possible. Other reasons why true non-contrast CT scans were additionally available to contrast-enhanced scans included the depiction of bone marrow pathologies as well as disk herniations or to visualize and further assess gout crystals or kidney stones. Patients with known dorsal instrumentation, spondylodiscitis, malignancy of the spine, and intervertebral spacers were excluded. After exclusion, the final data consisted of 91 patients. To prevent possible distortion of statistics in terms of time-related bias, only patients with a maximum examination interval of three weeks were included. Figure 1 illustrates the selection process in this study.

### Dual-energy CT scan protocol

Unenhanced and portal venous phase CT series were performed on a third-generation dual-source dual-energy CT scanner (Somatom Force; Siemens Healthineers). The system operates at different tube voltages using the standard protocol at our institution in daily clinical routine (tube A, 90 kVp and 220 mAs; tube B, Sn150 kVp [0.64-mm tin filter] and 138 mAs). Contrast agent was injected with a delay of 85 s in the contrast-enhanced portal venous phase. The injected contrast volume differed depending on body weight according to standard protocols in clinical routine. Patients were scanned in craniocaudal direction with the following device settings: gantry rotation time, 0.5 s; collimation, 128 × 0.6 mm; pitch, 0.6. Attenuation-based tube current was modulated automatically (CARE Dose 4D; Siemens Healthineers).

For unenhanced CT scans, the mean volume CT dose index was 8.6 mGy ± 3.5 (range, 5.2–11.4 mGy) and the mean dose-length product was 297.3 mGy · cm ± 68.2 (range, 209.7–379.8 mGy · cm). Portal venous phase CT scans generated a mean volume CT dose index of 13.6 mGy ± 3.4 (range, 10.6–17.2 mGy) and a mean dose-length product of 461.8 mGy · cm ± 65.9 (range, 336.9–571.2 mGy · cm).

### CT image reconstruction and postprocessing

After extracting unenhanced and portal venous phase image sets with 90 kVp and Sn150 kVp from our database, weighted average images were reconstructed at a ratio of 0.5:0.5 to simulate a single-energy 120 kVp image set, as described in previous publications [14, 18]. Reconstruction was performed using a medium-soft convolution kernel (Qr40) and advanced model-based iterative reconstruction software (ADMIRE, level of 3). Further postprocessing steps were conducted on a commercially available workstation (syngo.via, version VB10B; Siemens Healthineers). Imaging was based on a material decomposition algorithm optimized for a colored depiction of intervertebral disks. A dedicated approach for reconstruction of VNCa images was applied for the colored analysis of intervertebral disks using default software settings (color lookup table low-energy value, spectrum; color lookup table high-energy value, grayscale; CT preset 1, liver; CT preset 2, bone), as previously described by Booz et al. [14]. Reconstruction time for each VNCa image was noted. Finally, axial (section thickness of 2 mm and increment of 1 mm) and sagittal (section thickness and increment of 2 mm) images were created and sent to the picture archiving and communication system.

### MRI scan protocol

Native and contrast-enhanced MRI scans served as reference standard performed on a 3.0-T system (Magnetom PrismaFit; Siemens Healthineers) with a dedicated spine surface coil. The protocol of both phases consisted of a standard T1-weighted spin-echo sequence (repetition time msec/echo time msec, 650/10; matrix size, 288 × 384; section thickness, 4 mm), a T2-weighted fast spin-echo sequence (4000/89; matrix size, 358 × 448; section thickness, 4 mm), and a turbo inversion-recovery magnitude sequence (3500/39; matrix size, 388 × 384; section thickness, 4 mm).

### Image analysis

Image sets were evaluated on a standard picture archiving and communication system (Centricity, version 4.2; GE Healthcare), as previously described [14].

All MRI series were analyzed by one experienced radiologist (T.J.V., board-certified radiologist with 34 years of experience in musculoskeletal imaging) for the presence and degree of lumbar disk herniation to define the standard of reference. Analysis of disk herniations was based on the classification of the NASS [6]. The reader was blinded to clinical or CT data.

After establishing the reference standard, five readers (J.L.W., a board-certified radiologist with 10 years of experience; T.D., a board-certified radiologist with 8 years of experience; S.S.M., radiology resident with 8 years of experience; M.H.A., radiology resident with 6 years of experience; I.Y., radiology resident with 5 years of experience) independently evaluated dual-energy CT series, blinded to any data. First, colored-coded VNCa reconstructions derived from the unenhanced phase were analyzed for lumbar disk herniation on a per-disk basis. After 8 weeks, readers assessed colored VNCa reconstructions from the contrast-enhanced portal venous phase in the same way without access to data from the unenhanced phase.

For both, MRI and CT analyses, ratings for diagnostic confidence, image quality, and noise were performed individually using five-point Likert scales (scale range of 1–5: 1, unacceptable; 5, excellent). All readers could freely modify and adjust window settings, as well as the contrast level during the image evaluation in this study. This allowed for a certain amount of customization by otherwise standardized steps of VNCa reconstructions.

### Statistical analysis

Commercially available software was used for statistical analysis (SPSS Statistics for Windows, version 23.0; IBM, and MedCalc for Windows, version 13; MedCalc). The normality of data was assessed by the nonparametric Kolmogorov–Smirnov test. Variables were expressed as means ± standard deviation and analyzed with the Wilcoxon test. A *p* < 0.05 was considered statistically significant.

Sensitivity, specificity, and accuracy values were calculated on a per-disk basis. The obtained values were compared between colored VNCa series from unenhanced and contrast-enhanced portal venous phase CT scans by using the McNemar test for binary matched-pairs data. Clustering of lumbar disks was taken into account as previously described by Genders et al. [23]. Interrater agreement was evaluated by calculating weighted Fleiss *κ* according to Landis and Koch [24].

## Results

In total, 538 lumbar intervertebral disks (Th12-S1) (median per patient, 6; range, 5–6) in 91 patients (65 years ± 16; range, 26–92 years) consisting of 43 women (47%; 68 years ± 18; range, 26–92 years) and 48 men (53%; 63 years ± 17; range, 33–87 years) were finally included (Table 1). Eighteen patients were previously excluded owing to implanted osteosynthesis material (7 patients), malignancy of the spine (4 patients), spondylodiscitis (4 patients), and intervertebral spacers (3 patients). The most frequent CT indications were malignant melanoma (32%, *n* = 29), lymphoma (24%, *n* = 22), colorectal adenocarcinoma (18%, *n* = 16), hepatocellular carcinoma (13%, *n* = 12), infectious diseases (10%, *n* = 9), and ovarian carcinoma (3%, *n* = 3). Subsequent MRI examinations were conducted due to persistent lower back pain. MRI revealed a total of 210 lumbar herniated disks (39% of all evaluated lumbar intervertebral disks; median per patient, 3; range, 1–5). According to the classification of the NASS [25], lumbar disk herniations were classified as 123 protrusions (58%), 81 extrusions (39%), and 6 sequestrations (3%). The mean examination interval between dual-energy CT and MRI was 7 days (range, 0–19 days). The reconstruction time of VNCa images was 2 min on average in both CT series without significant difference (range, 1–3 min, *p* > 0.05).

**Table 1:** Patient population data (*n* = 91)

Characteristics	Value
Total number of patients (women; men)	91 (43; 48)
Overall mean age (y) ± SD, range	65 ± 16, 26–92
Overall mean BMI (kg/m^2^) ± SD, range	29 ± 3, 20–37
Mean age of women (y) ± SD, range (Mean BMI of women (kg/m^2^) ± SD, range)	68 ± 18, 26–92 (30 ± 4, 21–35)
Mean age of men (y) ± SD, range (Mean BMI of men (kg/m^2^) ± SD, range)	63 ± 17, 33–87 (28 ± 3, 20–37)
Number of patients with known lumbar disk herniation	6/91 (7%)
Number of patients with known osteoporosis	15/91 (17%)
Number of patients with known scoliosis	7/91 (8%)

*SD* standard deviation, *BMI* body mass index

### Diagnostic accuracy

**Fig. 2:**
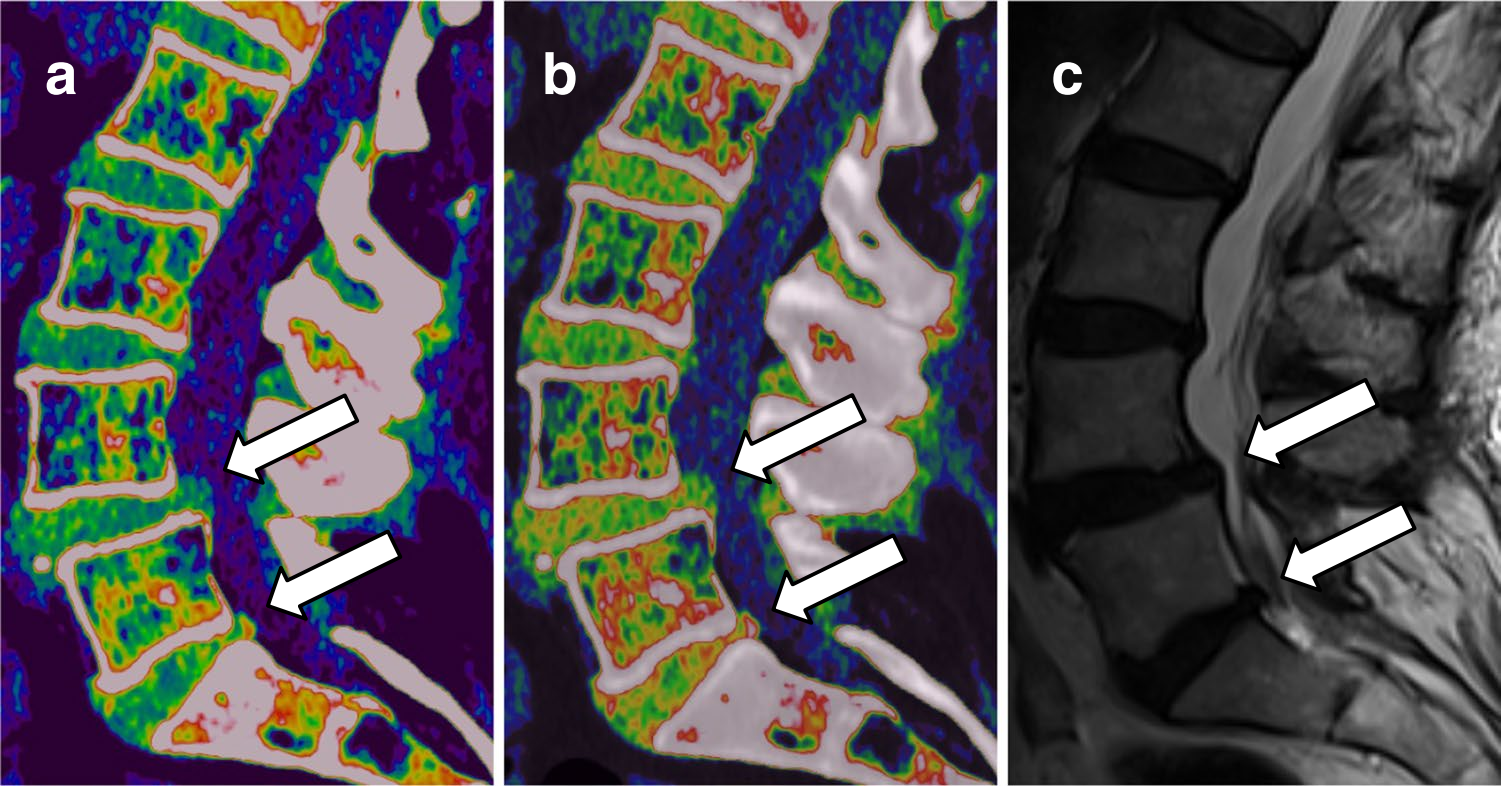
Case of a 61-year-old man with malignant melanoma who presented to the emergency department with severe lower back pain after lifting heavy weights at work. The patient initially underwent spine dual-energy CT imaging and afterwards MRI due to persistent lower back pain. Unenhanced sagittal VNCa reconstruction (**a**) depicted median lumbar disk protrusions at levels L4/5 and L5/S1 (*arrows*) and bulging of L3/4. Both, lumbar disk protrusions and bulging, were also clearly visible on sagittal VNCa CT series derived from contrast-enhanced portal venous phase (*arrows*) (**b**). T2-weighted MRI series confirmed the diagnosis of median lumbar disk protrusions at level L4/5 and L5/S1, as well as bulging of L3/4 on the sagittal plane (*arrows*) (**c**)

Analysis per intervertebral disk revealed comparable high overall sensitivity (93% [95% CI, 0.89–0.96] vs 95% [95% CI, 0.92–0.97]), specificity (94% [95% CI, 0.91–0.97] vs 95% [95% CI, 0.93–0.98]), and accuracy (94% [95% CI, 0.89–0.98] vs 95% [95% CI, 0.90–0.98]) of contrast-enhanced portal venous phase color-coded VNCa reconstructions for the assessment of lumbar disk herniation compared to unenhanced VNCa scans (all comparisons, *p* > 0.05) (Fig. 2).

**Fig. 3:**
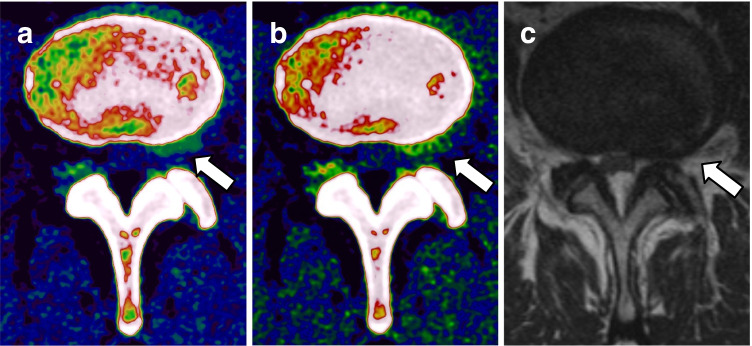
Case of a 68-year-old man suffering from lymphoma with ongoing lower back pain who underwent spine dual-energy CT imaging and MRI. Analysis of transversal color-coded VNCa images showed left-sided mediolateral lumbar disk protrusion at level L3/4 (*arrow*), which was detected on both, unenhanced (**a**) and contrast-enhanced portal venous phase (**b**) VNCa imaging by 5/5 readers. Axial T2-weighted MRI series confirmed the left-sided disk protrusion (*arrow*) (**c**)

Regarding lumbar disk protrusion, statistical analysis revealed similar high overall sensitivity (92% [95% CI, 0.89–0.95] vs 94% [95% CI, 0.92–0.97]), specificity (94% [95% CI, 0.91–0.97] vs 96% [95% CI, 0.93–0.99]), and accuracy (93% [95% CI, 0.91–0.98] vs 95% [95% CI, 0.92–0.98]) of VNCa reconstructions from contrast-enhanced portal venous phase and unenhanced CT series (all comparisons, *p* > 0.05) (Table 2). Interrater agreement was excellent for VNCa images derived from both, contrast-enhanced portal venous phase (*ϰ* = 0.86 [95% CI, 0.81––0.89]) and unenhanced CT series (*ϰ* = 0.84 [95% CI, 0.78–0.89]) (*p* > 0.05). Figure 3 shows a case with left-sided mediolateral lumbar disk protrusion at level L3/4, which was properly detected by all five readers on VNCa images derived from both phases.

**Table 2:** Diagnostic performance of color-coded VNCa reconstructions derived from unenhanced and contrast-enhanced portal venous phase CT series for the classification of lumbar disk herniation (no difference, *p* > .05)

Disk-based	Sensitivity	Specificity	Accuracy
Thoracic disk protrusion			
VNCa U	94% [0.92–0.97]	96% [0.93–0.99]	95% [0.92–0.98]
VNCa C	92% [0.89–0.95]	94% [0.91–0.97]	93% [0.91–0.98]
Thoracic disk extrusion			
VNCa U	92% [0.87–0.97]	94% [0.89–0.98]	93% [0.89–0.97]
VNCa C	94% [0.90–0.98]	95% [0.91–0.99]	94% [0.90–0.98]
Thoracic disk sequestration			
VNCa U	100%	100%	100%
VNCa C	100%	100% [0.98–1.00]	100% [0.99–1.00]

*VNCa* virtual noncalcium, *U* unenhanced, *C* contrast-enhanced. Values in square brackets are 95% confidence intervals

The ratings of each CT reader differed not significantly in terms of sensitivity, specificity, and accuracy (all comparisons, *p* > 0.05). The most experienced reader (J.L.W.) yielded an almost perfect match with MRI as reference standard in assessing lumbar disk herniation with an overall sensitivity of 97% (95% CI, 0.94–0.98), specificity of 98% (95% CI, 0.95–0.99), and accuracy of 98% (95% CI, 0.95–0.99) (Table 3).

**Table 3:** Reader-based illustration of diagnostic accuracy for the detection of lumbar disk herniation per intervertebral disk**.** Diagnostic accuracy of all readers remained high in contrast-enhanced portal venous phase VNCa reconstructions. No interrater differences were observed (*p* > .05). Experience level in musculoskeletal imaging: reader 1, 10 years; reader 2, 8 years; reader 3, 8 years; reader 4, 6 years; and reader 5, 5 years

			Sensitivity		Specificity	Accuracy
Average	VNCa U			95% [0.92–0.97]	95% [0.93–0.98]	95% [0.90–0.98]
	VNCa C			93% [0.89–0.96]	94% [0.91–0.97]	94% [0.89–0.98]
Reader 1	VNCa U			96% [0.94–0.97]	96% [0.94–0.98]	96% [0.93–0.98]
	VNCa C			97% [0.94–0.98]	98% [0.95–0.99]	98% [0.95–0.99]
Reader 2	VNCa U			94% [0.91–0.97]	96% [0.93–0.98]	95% [0.92–0.98]
	VNCa C			94% [0.90–0.97]	94% [0.90–0.98]	94% [0.90–0.98]
Reader 3	VNCa U			95% [0.91–0.98]	96% [0.94–0.98]	96% [0.93–0.97]
	VNCa C			92% [0.89–0.95]	95% [0.92–0.98]	94% [0.91–0.96]
Reader 4	VNCa U			90% [0.88–0.92]	93% [0.90–0.96]	92% [0.89–0.95]
	VNCa C			92% [0.89–0.95]	94% [0.91–0.97]	93% [0.91–0.95]
Reader 5	VNCa U			91% [0.86–0.96]	90% [0.86–0.93]	91% [0.87–0.94]
	VNCa C			93% [0.89–0.97]	93% [0.90–0.95]	93% [0.90–0.96]

*VNCa* virtual noncalcium, *U* unenhanced, *C* contrast-enhanced. Values in square brackets are 95% confidence intervals

An additional subanalysis on a small patient cohort (*n* = 11) with pronounced degenerations of the lumbar spine revealed comparable high overall diagnostic accuracy for the assessment of lumbar disk herniation (91% [95% CI, 0.87–0.96] vs 92% [95% CI, 0.88–0.97]) of color-coded VNCa reconstructions derived from contrast-enhanced portal venous phase and unenhanced CT scans (*p* > 0.05).

### Image ratings

**Fig. 4:**
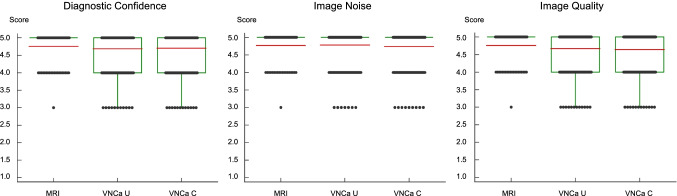
Image ratings regarding diagnostic confidence, image noise, and quality of MRI and color-coded VNCa series derived from unenhanced (VNCa U) and contrast-enhanced portal venous phase (VNCa C) CT series. Mean scores are depicted as horizontal red lines and dots represent the distribution of scores. Ratings differed not significantly between MRI series and unenhanced or contrast-enhanced portal venous phase VNCa reconstructions (all *p* > .05)

The experienced single reader demonstrated high diagnostic confidence (4.76 ± 0.46) in assessing lumbar disk herniation on MRI series. Diagnostic confidence of color-coded VNCa images from contrast-enhanced portal venous phase and unenhanced CT series (4.71 ± 0.50 and 4.69 ± 0.51) revealed no significant differences (*p* = 0.55) (Fig. 4). Interrater agreement was excellent for contrast-enhanced portal venous phase VNCa images (*ϰ* = 0.87 [95% CI, 0.84–0.90]) and unenhanced VNCa images (*ϰ* = 0.89 [95% CI, 0.86–0.92]).

Image quality was rated with mean scores of 4.76 ± 0.46 for MRI, 4.65 ± 0.52 for contrast-enhanced portal venous phase VNCa reconstructions, and 4.67 ± 0.51 for unenhanced VNCa reconstructions without significant differences between the modalities (*p* = 0.45). In this context, interrater agreement was excellent for contrast-enhanced and unenhanced CT series, ranging from 0.86 (95% CI, 0.83–0.89) to 0.92 (95% CI, 0.88–0.96).

Image noise differed not significantly between MRI series (4.77 ± 0.45) and contrast-enhanced portal venous phase VNCa reconstructions (4.74 ± 0.47, *p* = 0.63), or unenhanced VNCa reconstructions (4.78 ± 0.44, *p* = 0.77). Again, interrater agreement was excellent for contrast-enhanced portal venous phase VNCa images (*ϰ* = 0.87 [95% CI, 0.84–0.90]) and unenhanced VNCa images (*ϰ* = 0.89 [95% CI, 0.82–0.96]).

## Discussion

Our study results demonstrated for the first time that color-coded VNCa reconstructions derived from contrast-enhanced portal venous phase dual-energy CT yield similar diagnostic accuracy for the depiction of lumbar disk herniation compared to unenhanced VNCa imaging. Diagnostic confidence, image quality, and noise levels differed not significantly between unenhanced and contrast-enhanced VNCa imaging (all *p* > 0.05). Concomitantly, VNCa reconstructions derived from unenhanced and portal venous phase CT series achieved high levels of interrater agreement, pointing towards great diagnostic reliability of this recently developed dual-energy CT postprocessing algorithm also in contrast-enhanced CT series.

Previous studies on unenhanced color-coded VNCa imaging demonstrated high diagnostic performance and confidence for the visualization of cervical, thoracic, and lumbar disk herniation including associated spinal nerve root impingement compared to conventional grayscale CT [13, 14, 22]. The substantially different architecture of each spinal section had no effect on the diagnostic capability of the proposed approach for reconstruction of VNCa images to depict intervertebral disk herniation [10, 26, 27]. However, to the best of our knowledge, no studies have investigated the impact of contrast agent on the visualization of lumbar disk herniation using color-coded VNCa imaging.

Our study results underline the potential of VNCa reconstructions derived from dual-source dual-energy CT to serve as a valuable imaging alternative to MRI also in the case of contrast-enhanced portal venous phase imaging. In this context, all readers demonstrated comparable high diagnostic accuracy and confidence for the accurate detection of herniated disks without significant differences in comparison to VNCa images derived from unenhanced CT scans. Regarding the correct graduation of lumbar disk herniations according to the current clinical guidelines of the NASS [25], contrast agent did not influence the capability of VNCa reconstructions to depict even relatively small protrusions or extrusions. VNCa imaging derived from contrast-enhanced portal venous phase CT series allowed for excellent demarcation of lumbar disks from cerebrospinal fluid. Lumbar disk sequestrations were identified in all cases resulting in a diagnostic accuracy of 100% in both, unenhanced and contrast-enhanced VNCa series. We observed a somewhat lower diagnostic accuracy of color-coded VNCa reconstructions in patients with marked lumbar spine degenerations in comparison to younger patients without or only moderate degenerations due to overlap by spondylophytes in close proximity to lumbar disks. An additional subanalysis on a small patient cohort (*n* = 11) showing pronounced degenerations of the lumbar spine revealed comparable high overall diagnostic accuracy for the assessment of lumbar disk herniation (91% [95% CI, 0.87–0.96] vs 92% [95% CI, 0.88–0.97]) of color-coded VNCa reconstructions derived from contrast-enhanced phase and unenhanced VNCa scans (*p* > 0.05). Future studies should focus on optimized postprocessing algorithms to achieve a better differentiation of lumbar disk herniations from adjacent osteophytic alterations. In preliminary investigations to achieve optimal settings of the proposed postprocessing algorithm, our study group extensively tested possible alterations through the application of different kV. Finally, the applied kV values of dual-source dual-energy CT in combination with dedicated postprocessing settings represent the most appropriate setup for lumbar disk herniation detection based on VNCa reconstructions in our opinion. Interestingly, body mass index did not influence the ability of VNCa reconstructions to detect lumbar disk herniations. In practical terms, VNCa image reconstruction took only 2 minutes on average (range, 1–3 minutes) without significant differences between unenhanced and contrast-enhanced portal venous phase image series, pointing towards a time-efficient integrability into daily clinical routine.

Our study findings are important to eliminate uncertainties arising from the application of VNCa reconstructions in contrast-enhanced portal venous phase CT scans, particularly in light of the fact that the majority of clinically performed examinations are conducted by using contrast agent, mainly in the context of oncological staging examinations and vascular or infectious diseases [10, 28, 29]. The shown high diagnostic accuracy of VNCa reconstructions based on contrast-enhanced portal venous phase dual-energy CT scans emphasizes its great potential for retrospective opportunistic lumbar disk herniation assessment in examinations performed for other indications, which may result in earlier diagnosis and rapid therapy initiation as well as prevention of additional examinations in clinical routine. In this context, a diagnosed lumbar disk herniation can be treated in most cases conservatively, leading to recovery of about one-third of patients within two weeks after diagnosis [30]. However, establishing a prompt diagnosis is especially important in cases with compression of adjacent neural structures by herniated disks, otherwise potentially resulting in progressive radiculopathy or myelopathy with symptoms such as pain, numbness, weakness, or even incontinence [1]. Regarding such complicated cases, discectomy has been shown to provide faster pain relief and recovery from disability than conservative treatment in several randomized controlled trials [31, 32]. Ultimately, the application of VNCa imaging potentially may reduce the substantial social and economic burden associated with this common degenerative disorder. In addition, the results of this study emphasize that color-coded VNCa reconstructions derived from contrast-enhanced portal venous phase dual-source dual-energy CT series as well as from unenhanced images may serve as a viable imaging alternative for the detection of lumbar disk herniation in case of MRI unavailability (e.g., in small hospitals with limited availability of MRI scanners) or contraindications, potentially improving the flexibility in clinical routine.

Several limitations of this study have to be addressed. First, the results of our study were obtained with a vendor-specific CT system and corresponding postprocessing software. This limits the transferability of our findings to CT systems merchandised by other vendors. Second, contrast volume differed between performed CT examinations with varying volume and flow rate of contrast agent. However, our findings underline the potential of contrast-enhanced VNCa reconstructions derived from dual-source dual-energy CT for an accurate depiction of lumbar disk herniation, despite possible contrast agent variations. Third, the retrospective single-center study design has led to a limited number of available study patients. Additionally, clinical data including outcome was not evaluated in this study. Therefore, forthcoming studies on big patient populations including clinical data are needed to validate the preliminary findings of our study and also to further analyze the clinical value of this CT technique. Fourth, MRI may have caused possible overestimation of disk herniation classification [33, 34]. Fifth, the proposed postprocessing algorithm was specifically designed to assess disk herniations and optimized for the best demarcation of cerebrospinal fluid and soft disk herniations. Therefore, analysis of Modic changes was not included in our study. Other dedicated software settings allow for a colored visualization of bone marrow alterations such as bone marrow edema or fatty bone degeneration, which would be partially necessary for CT-based assessment of Modic changes [35, 36]. A combined analysis of both algorithms for disc and bone marrow assessment should be object of future investigations. Finally, the diagnostic accuracy of VNCa imaging for assessing disk herniation was only evaluated in the unenhanced and contrast-enhanced portal venous phase. Future research should also focus on whether other contrast-enhanced phases affect the diagnostic capability of VNCa reconstructions.

In conclusion, this study showed that color-coded VNCa reconstructions derived from contrast-enhanced portal venous phase dual-source dual-energy CT yield similarly high diagnostic accuracy for assessing lumbar disk herniation compared to VNCa reconstructions derived from unenhanced CT series. Moreover, ratings for diagnostic confidence, image quality, and noise scores were equivalent between unenhanced and contrast-enhanced portal venous phase VNCa reconstructions. Therefore, dual-source dual-energy CT-based VNCa imaging does not only allow for highly accurate opportunistic retrospective lumbar disk herniation assessment in unenhanced CT scans but also in portal venous phase CT series, which represent the most frequently applied phase in tumor staging examinations or scans performed in the context of vascular or infectious diseases. Ultimately, application of this technique may result in earlier diagnosis and rapid therapy initiation as well as prevention of additional examinations in daily clinical routine, potentially leading to reduction of the substantial social and economic burden associated with this common degenerative disorder.

## Abbreviations

CI: Confidence interval
VNCa: Virtual noncalcium

## Declarations

### Guarantor

The scientific guarantor of this publication is Prof. Dr. Vogl.

### Statistics and biometry

No complex statistical methods were necessary for this paper.

### Methodology

• Retrospective

• Diagnostic or prognostic study

• Performed at one institution
